# Nonlinear Maximization of the Sum-Frequency Component from Two Ultrasonic Signals in a Bubbly Liquid

**DOI:** 10.3390/s20010113

**Published:** 2019-12-23

**Authors:** María Teresa Tejedor Sastre, Christian Vanhille

**Affiliations:** NANLA, Departamento de Matemática Aplicada, Ciencia e Ingeniería de los Materiales y Tecnología Electrónica, Universidad Rey Juan Carlos, Tulipán s/n, 28933 Móstoles, Madrid, Spain; mariateresa.tejedor@urjc.es

**Keywords:** bubbly liquids, nonlinear acoustics, numerical models, nonlinear frequency mixing, sum-frequency component, nonlinear resonance

## Abstract

Techniques based on ultrasound in nondestructive testing and medical imaging analyze the response of the source frequencies (linear theory) or the second-order frequencies such as higher harmonics, difference and sum frequencies (nonlinear theory). The low attenuation and high directivity of the difference-frequency component generated nonlinearly by parametric arrays are useful. Higher harmonics created directly from a single-frequency source and the sum-frequency component generated nonlinearly by parametric arrays are attractive because of their high spatial resolution and accuracy. The nonlinear response of bubbly liquids can be strong even at relatively low acoustic pressure amplitudes. Thus, these nonlinear frequencies can be generated easily in these media. Since the experimental study of such nonlinear waves in stable bubbly liquids is a very difficult task, in this work we use a numerical model developed previously to describe the nonlinear propagation of ultrasound interacting with nonlinearly oscillating bubbles in a liquid. This numerical model solves a differential system coupling a Rayleigh–Plesset equation and the wave equation. This paper performs an analysis of the generation of the sum-frequency component by nonlinear mixing of two signals of lower frequencies. It shows that the amplitude of this component can be maximized by taking into account the nonlinear resonance of the system. This effect is due to the softening of the medium when pressure amplitudes rise.

## 1. Introduction

Ultrasound-based techniques have been widely used in material science to characterize the medium or to localize cracks (nondestructive evaluation) [1,2,3,4] and in medical imaging to diagnose pathologies [5,6]. These techniques rely on the detection of transmitted or reflected ultrasonic waves. They use the signals emitted from the source (primary frequencies) during the entire process [6], which falls under the linear theory valid for infinitesimal pressure amplitudes, or consider combinations of the source signals such as harmonics, difference frequencies, sum frequencies, subharmonics [7,8,9,10,11], which falls under the nonlinear theory (at least second-order terms) valid for finite pressure amplitudes. The benefits of the signal produced at the difference frequency by parametric arrays stand in its low attenuation and high directivity. This gives rise to a high penetration of the information transmitted through the medium [12,13]. In addition to the difference-frequency field, the nonlinear combination of two signals of different frequencies emitted at finite amplitudes also produce the sum-frequency component. The wavelength of this sum-frequency signal is shorter than the ones of the source and difference frequencies. This particularity is very attractive when a high resolution or accuracy of the results is required, as for example in medical imaging or nondestructive testing [10,11].

**Figure 1:**
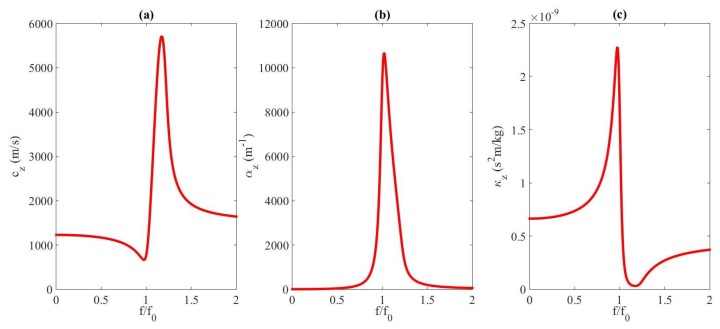
Curves of dispersion in a bubbly liquid for sound speed $c_{z}$ (**a**), attenuation coefficient $\alpha_{z}$ (**b**), and compressibility coefficient $\kappa_{z}$ (**c**).

Liquids containing gas bubbles are very interesting media since a tiny amount of void fraction modifies the acoustic properties of the medium drastically, introducing dispersion, attenuation, and nonlinearity. The sound speed, attenuation coefficient, and parameter of nonlinearity are dependent on the ratio of their frequency, *f*, to the resonance frequency of the bubble, $f_{0}$. They can change by orders of magnitude from one signal frequency to another [14,15]. Figure 1 illustrates the dispersive character of a bubbly medium by representing the sound speed $c_{z}$ (a), attenuation coefficient $\alpha_{z}$ (b), and compressibility coefficient $\kappa_{z}$ (c) vs. $f/f_{0}$ obtained from the development given in Refs. [14,15]. The liquid with bubbles shown here is the one used in the simulations performed in Section 3. It must be noted that this frequency dependence is similar for any other medium made of liquid and gas bubbles. The variations of these parameters with the frequency are very large. Their influence on the propagation of ultrasound is huge. Thus, at some frequency ranges, the very high nonlinearity of the medium due to the presence of bubbles is responsible for the generation of higher harmonics, difference and sum frequencies even at moderate finite-pressure amplitudes [15,16,17]. Bubbles are widely used as contrast agents in medical imaging [18].

The control of the numerous parameters which come into play in the set-up of experimental systems to study the behavior of finite-amplitude ultrasound propagating in liquids with a stable population of gas bubbles is a very difficult task. The development of numerical models able to approximate the nonlinear response of such systems is thus necessary to analyze the generation of harmonics, difference and sum frequencies during the propagation of finite-amplitude acoustic waves in bubbly liquids [19,20,21,22,23].

The decrease of sound speed when acoustic amplitudes are raised has been previously observed in several nonlinear and dispersive media by evidencing the nonlinear shift of the resonance frequency of the system [24,25,26]. The drop of sound speed is associated to the softening of the medium when pressure amplitudes are raised. In particular, it was studied to maximize the fundamental and difference-frequency components in a bubbly liquid [27]. However, up to our knowledge, this behavior has not been analysed for the sum-frequency component. In this paper, we study whether this frequency shift effect is observable by analysing the sum-frequency component obtained by nonlinear mixing of two signals of lower frequencies. We also investigate the possibility of taking advantage of this frequency shift to maximize the sum-frequency component amplitude, which could be useful to improve the resolution or accuracy of the response of the system. This improvement may be of interest in applications of nonlinear acoustics and nondestructive testing [28,29,30,31].

In Section 2 we describe the physical problem studied here and the mathematical system we consider to model the nonlinear interaction of ultrasound and bubbles. Section 3 reports the numerical simulations performed and the results of this study. In Section 3.1 we compare the efficacy of the nonlinear generation of higher frequency components from one or two primary frequencies at the source. The study of the nonlinear generation of the sum-frequency component by mixing two source frequencies is then analysed using a linear resonance assumption in Section 3.2. The analysis of the change of sound speed and cavity resonance for the sum-frequency component vs. acoustic pressure amplitudes is performed in Section 3.3. The nonlinear generation of the sum-frequency component is thus studied by mixing two source frequencies and considering a nonlinear resonance assumption in Section 3.4. Finally, the results of this work are discussed in Section 4, in which the enhancement of the sum-frequency signal obtained from nonlinear frequency mixing by considering the nonlinear resonance effect is shown.

## 2. Materials and Methods

We considered an ultrasonic field traveling through a mixture of water and a high density of tiny air bubbles evenly distributed in a one-dimensional cavity of length *L*.

The nonlinear interaction between the acoustic field p(x,t) and the bubble oscillations, expressed in volume variation, $v\left(x,t\right)=V\left(x,t\right)-v_{0g}$, can be described by the system of differential equations formed by the wave Equation (1) and a Rayleigh–Plesset Equation (2) [14,16,17], where *x* is the one-dimensional space coordinate, *t* is the time, $T_{t}$ is the last instant of the study, *V* is the current volume of bubble, and $v_{0g}=\frac{4}{3}\pi R_{0g}^{3}$ is the initial bubble volume, with initial radius $R_{0g}$:

(1) $$\frac{\partial^{2}p}{\partial x^{2}}-\frac{1}{c_{0l}^{2}}\frac{\partial^{2}p}{\partial t^{2}}=-\rho_{0l}N_{g}\frac{\partial^{2}v}{\partial t^{2}},\;0<x<L,\;0<t<T_{t},$$

(2) $$\frac{\partial^{2}v}{\partial t^{2}}+\delta \omega_{0g}\frac{\partial v}{\partial t}+\omega_{0g}^{2}v+\eta p=av^{2}+b\left(2v\frac{\partial^{2}v}{\partial t^{2}}+{\left(\frac{\partial v}{\partial t}\right)}^{2}\right),\;0\leq x\leq L,\;0<t<T_{t}.$$

In Equation (1) $c_{0l}$ and $\rho_{0l}$ are the sound speed and the density at the equilibrium state of the liquid, and $N_{g}$ is the density of bubbles in the liquid. In Equation (2) $\delta =4\nu_{l}/\omega_{0g}R_{0g}^{2}$ is the viscous damping coefficient of the bubbly fluid, in which $\nu_{l}$ is the cinematic viscosity of the liquid, $\omega_{0g}=\sqrt{3\gamma_{g}p_{0g}/\rho_{0l}R_{0g}^{2}}$ is the resonance frequency of the bubbles, in which $\gamma_{g}$ is the specific heats ratio of the gas, $p_{0g}=\rho_{0g}c_{0g}^{2}/\gamma_{g}$ is its atmospheric pressure, and $\rho_{0g}$ and $c_{0g}$ are the density and sound speed at the equilibrium state of the gas. The parameter $\eta =4\pi R_{0g}/\rho_{0l}$ is a constant. $a=\left(\gamma_{g}+1\right)\omega_{0g}^{2}/2v_{0g}$ and $b=1/6v_{0g}$ are the nonlinear coefficients corresponding to the adiabatic gas law and to the dynamic of bubbles. This differential system models the nonlinear interaction of ultrasound and bubble oscillations, which causes attenuation and dispersion in the bubbly liquid. Note that both dependent variables, *p* and *v*, are unknown in the system. Therefore, they are both nonlinear, the distortion of one of them affects the other one and vice versa. The model also accounts for approximations which idealize the physical problem. Among others, all the bubbles are spherical and of the same size, $f_{0}$ corresponds to their lowest mode, adiabatic gas law is assumed in the bubble, bubbles do not radiate sound themselves, the buoyancy, viscous drag, and Bjerknes forces are not considered [15].

The auxiliary conditions are the followings. At the onset of the study we considered

(3) $$p\left(x\neq 0,0\right)=0,v\left(x,0\right)=0,\;\frac{\partial p}{\partial t}\left(x\neq 0,0\right)=0,\;\frac{\partial v}{\partial t}\left(x,0\right)=0,\;0\leq x\leq L.$$

The cavity was excited by a time-dependent pressure source s(t) placed at x=0:

(4) $$p\left(x=0,t\right)=s\left(t\right),\;0\leq t\leq T_{t},$$

and we assumed a free-walled boundary condition at x=L:

(5) $$p\left(L,t\right)=0,\;0\leq t\leq T_{t}.$$

This differential system, Equations (1)–(5), is solved using the numerical model developed in Ref. [21]. It is based on the finite-volume method in the space dimension and on the finite-difference method in the time dimension. 64 finite volumes have been used in the cavity and 400 time points are employed per period of the sum frequency $f_{s}$ in Section 3. In each of them a discretized version of Equations (1) and (2) is solved [21].

Whereas in this study the same numerical model as in Ref. [21] was used, our objective here was to analyze a different phenomenon. In Ref. [21] the generation of harmonics from a single-frequency source and the generation of the difference frequency $f_{d}$ from a dual-frequency source were studied. Ref. [27] was dedicated to the generation and optimization of the difference-frequency component $f_{d}$ from two primary signals of higher frequencies, whereas in this work the optimization was about $f_{s}$ obtained from two primary signals of lower frequencies. Whereas $f_{d}$ is less attenuated and can propagate over a larger distance, $f_{s}$ gives information with better precision. This latter point justifies the interest of this work. Refs. [11,23] studied the generation of $f_{s}$ from two primary signals only (which can be useful due to its high spatial resolution and accuracy), but here we also analyzed its maximization. The results of this work showed that we could obtain much higher amplitudes of $f_{s}$ by slightly reducing one of the primary frequencies (see Section 3).

## 3. Results

The data used for the simulations in this section are $c_{0l}=1500\;m/s$, $\rho_{0l}=1000\;{\mathrm{kg}/m}^{3}$, $\nu_{l}=1.43\times 10^{-6}\;m^{2}/s$ for the liquid (water) and $c_{0g}=340\;m/s$, $\rho_{0g}=1.29\;{\mathrm{kg}/m}^{3}$, $\gamma_{g}=1.4$ for the gas (air). We use bubbles of radius $R_{0g}=2.5\;\mu m$ and the bubble density is $N_{g}=5\times 10^{11}\;m^{-3}$. In all the experiments presented here the standing wave is formed in the resonator long before the last time $t=T_{t}$ is reached. The length of the cavity used in this section is defined by setting the half-wavelength resonance at 200kHz, far below the bubble resonance, and considering the sound speed $c_{z}$ evaluated from Refs. [14] (see also [15]) $c_{z200\;\mathrm{kHz}}=1222.8\;m/s$, i.e., $L=\lambda_{200\;\mathrm{kHz}}/2=c_{z200\;\mathrm{kHz}}/\left(2\left(200\;\mathrm{kHz}\right)\right)=0.0031\;m$.

### 3.1. Sum-Frequency Generation by Nonlinear Frequency Mixing vs. Harmonic Generation

**Figure 2:**
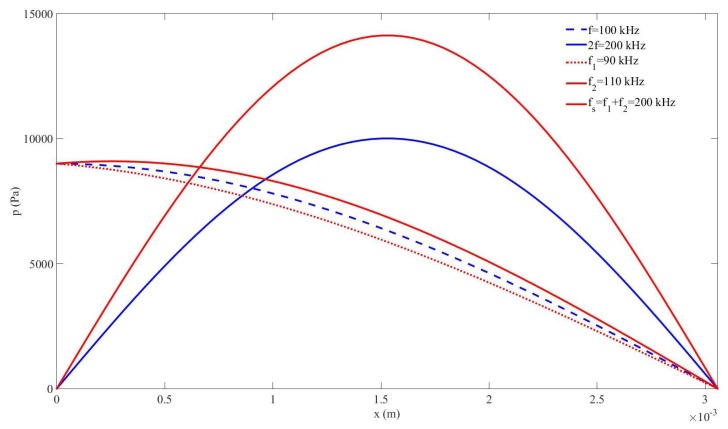
Pressure amplitude distribution of frequency components in the cavity; Case#1: blue (one source frequency), Case#2: red (two source frequencies); Source frequencies: dashed lines (fundamental and primary frequencies), nonlinear frequencies: solid lines (2nd harmonic and sum frequency).

The propagation of a high-frequency ultrasonic signal created from others of lower frequencies is certainly interesting in many frameworks, since it can be useful for obtaining a better spatial resolution in imaging or non destructive testing from transducers of lower frequencies. Based on nonlinear acoustics, two techniques are possible to achieve this task: (i) using a monochromatic source to create higher harmonics, with $s\left(t\right)=p_{0}sin\left(\omega t\right)$, where ω=2πf (Case#1), (ii) using a dual-frequency source to generate the sum-frequency $f_{s}$ by nonlinear frequency mixing, with $s\left(t\right)=p_{0}\left(sin\left(\omega_{1}t\right)+sin\left(\omega_{2}t\right)\right)$, where $\omega_{1}=2\pi f_{1}$ and $\omega_{2}=2\pi f_{2}$ (Case#2). In this section we compare the nonlinear generation of the same frequency $2f=f_{s}=f_{1}+f_{2}=200\;\mathrm{kHz}$ using both techniques in the nonlinear bubbly medium by setting the source at the same amplitude $p_{0}=9\;\mathrm{kPa}$ in both cases. The source frequencies are (i) f=100kHz for Case#1 and (ii) $f_{1}=90\;\mathrm{kHz}$ and $f_{2}=110\;\mathrm{kHz}$ for Case#2. $c_{zfs}=c_{2f}=1222.8\;m/s$. Figure 2 shows the amplitude of the frequency distribution of the acoustic pressure components in the cavity obtained after applying a fast Fourier transform. Blue and red colors correspond to Case#1 and Case#2, respectively. For Case#1, the fundamental (dashed line) and the second harmonic (solid line) are displayed. The maximum amplitude obtained for the second harmonic was $p_{2fm}=10.01\;\mathrm{kPa}$. For Case#2, the primary frequencies (dashed lines) and the sum frequency (solid line) are displayed. The maximum amplitude obtained for the sum frequency was $p_{fsm}=14.016\;\mathrm{kPa}$. The generation of the signal at 200kHz was more efficient by nonlinear frequency mixing (Case#2). This result justifies the following study in the next sections, which aims to enhance this signal by searching for its magnificence through nonlinear resonance.

### 3.2. Sum-Frequency Generation by Nonlinear Frequency Mixing under Linear Resonance Assumption

**Figure 3:**
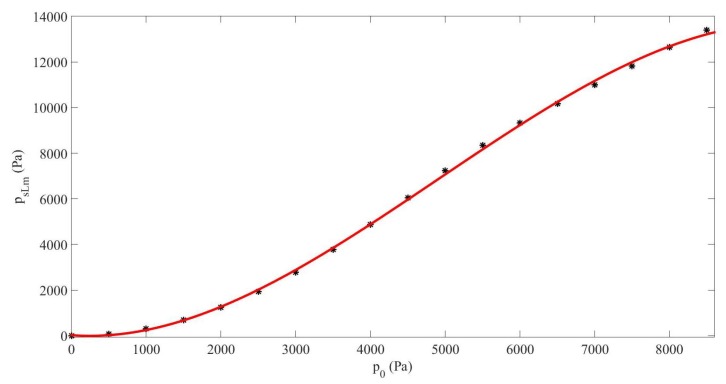
Case#2. Maximum amplitude of the sum-frequency pressure component in the cavity $p_{sLm}$ vs. source amplitude $p_{0}$ under linear resonance assumption (dots), and fitting curve (solid line).

In this section, and in the followings, we consider the source $s\left(t\right)=p_{0}\left(sin\left(\omega_{1L}t\right)+sin\left(\omega_{2L}t\right)\right)$ with the primary frequencies $f_{1L}=90\;\mathrm{kHz}$ and $f_{2L}=110\;\mathrm{kHz}$. As said in Section 3.1, the sum frequency obtained from Case#2, $f_{sL}=200\;\mathrm{kHz}$, travels in this medium at the sound speed $c_{fsL}=c_{z200\;\mathrm{kHz}}=1222.8\;m/s$, and we set the cavity length to be (linearly) resonant at $L=c_{fsL}/2f_{sL}=0.0031\;m$, i.e., for which the resonance was determined in the linear regime. In this configuration we studied the generation of the sum-frequency in the resonator as a function of the source amplitude $p_{0}$, but keeping the same sound speed, i.e., the same cavity length, for any of these amplitudes (sound speed at linear regime) [14,15]. Figure 3 shows the response of the system by displaying the maximum pressure amplitude of the sum-frequency found in the cavity $p_{sLm}$ vs. $p_{0}$. The result expressed in Pa fits to the following third-order polynomial, $p_{sLm}=-3.4\times 10^{-8}p_{0}^{3}+5\times 10^{-4}p_{0}^{2}-0.24p_{0}+25$. It must be noticed that the behavior given by this polynomial was valid in the range of $p_{0}$ employed here. The generation of the component $f_{sL}$ vs. $p_{0}$ was clearly nonlinear. Its amplitude increased rapidly in the middle range of $p_{0}$ and then quite moderately for higher amplitudes, due to the negative third-order coefficient in the polynomial, $-3.4\times 10^{-8}p_{0}^{3}$, which means that the amplitudes obtained were not excessively high for elevated $p_{0}$ values. The relative increase of amplitude generation of the component $f_{sL}$ in relation to $p_{0}$ was around 155.7% when $p_{0}=9\;\mathrm{kPa}$.

### 3.3. Nonlinear Resonance of the Cavity

**Figure 4:**
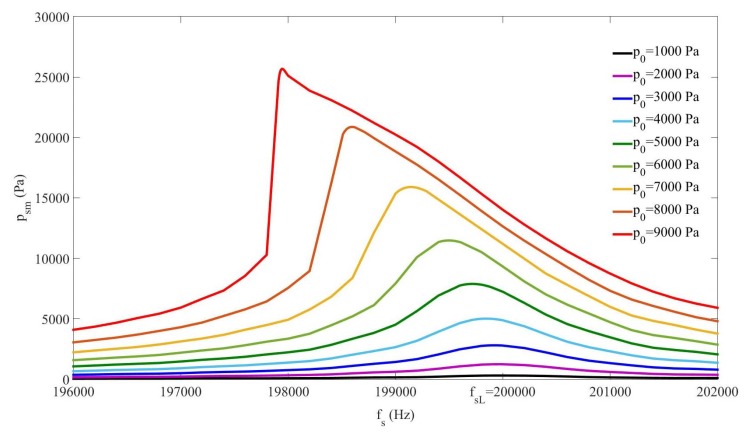
Case#2. Maximum amplitude of the sum-frequency pressure component in the cavity $p_{sm}$ vs. frequency $f_{s}$ (around $f_{sL}$) for different source amplitudes $p_{0}$.

It is well known that an increase of the pressure amplitude of an acoustic wave propagating in a bubbly liquid produces a softening of the medium, i.e., a decrease of the sound speed, which induces a shift of the resonance of the cavity (nonlinear frequency shift of the cavity resonance, nonlinear resonance) [24,25,26,27]. In this section we studied whether this effect could be detected and evaluated through the analysis of the sum frequency created by nonlinear mixing of two signals of lower frequencies (Case#2). We thus analyzed the resonance of the sum-frequency component of the signal pressure in the cavity by changing the amplitude at the source. For each amplitude, we applied a frequency sweep of $f_{s}$ around the linear resonance $f_{sL}=200\;\mathrm{kHz}$ (Section 3.2) by moving $f_{1L}$ by increment $\delta f_{1}=10\;\mathrm{Hz}$, whereas $f_{2L}=110\;\mathrm{kHz}$ remained constant during the whole procedure, to evaluate the highest pressure amplitude of the sum-frequency component reached in the cavity, $p_{sm}$, at each frequency. These $p_{sm}$ values are represented in Figure 4 for all the source amplitudes used in the study. The maximal value obtained over the entire frequency range at each amplitude was then localized, which defined the frequency for which the response of the system was maximum at this amplitude. The results show that when the amplitude increased, the frequency which maximized the response shifted towards lower frequencies, which means that the sound speed decreased, i.e., the medium softened. For example, with the source amplitude $p_{0}=9\;\mathrm{kPa}$ the frequency that gave the highest response, i.e., the “nonlinear-regime” resonance, was $f_{sm}=197.94\;\mathrm{kHz}$, corresponding to the sound speed $c_{fsNL}=2Lf_{sm}=1210.2\;m/s$, which was $\Delta f_{s}=2.06\;\mathrm{kHz}$ lower than the “linear-regime”resonance, 200kHz, obtained with low amplitudes. Although the change of sound-speed value was not very high, the variation of amplitude of the sum-frequency component was huge since it improved by 183.15%, from 14.016kPa (155.7% of the source amplitude) to 25.67kPa (285.3% of the source amplitude).

**Figure 5:**
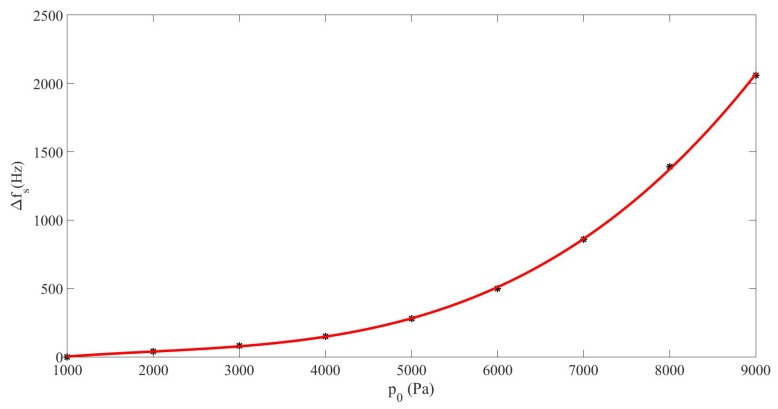
Case#2. Frequency shift of the sum-frequency pressure component $\Delta f_{s}$ vs. maximum sum-frequency pressure amplitude in the cavity $p_{sNLm}$ (dots), and fitting curve (solid line).

The shift $\Delta f_{s}$ undergone by the resonance frequency of the cavity was dependent on the maximum pressure amplitude reached in the cavity, corresponding to the sum-frequency component, $p_{sNLm}$, which depended on the source amplitude $p_{0}$, as it can be seen in Figure 5. It fit to the third-order polynomial $\Delta f_{s}=5.1\times 10^{-9}p_{0}^{3}-3\times 10^{-5}p_{0}^{2}+0.09p_{0}-63$, where pressure amplitudes are expressed in Pa and frequencies in Hz. The nonlinear behavior of this shift indicates that it rose very fast with $p_{0}$ since the third-order term was positive, $5.1\times 10^{-9}p_{0}^{3}$. This fact proves that the softening of the medium increased quite rapidly, i.e., the sound speed decreased fast, as $p_{0}$ was raised.

**Figure 6:**
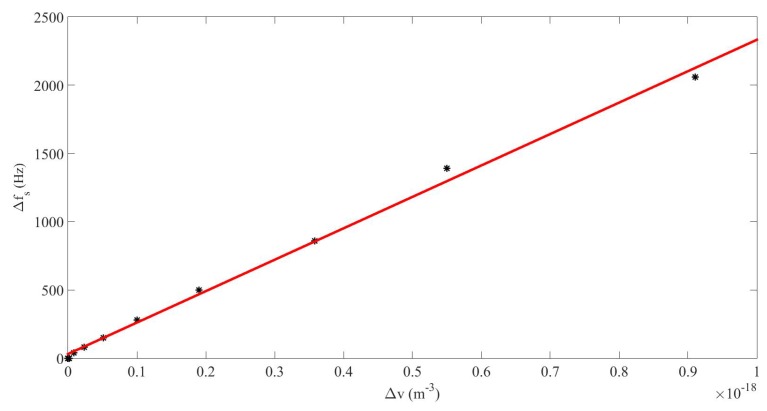
Case#2. Frequency shift of the sum-frequency pressure component $\Delta f_{s}$ vs. average bubble volume increase Δv (dots), and fitting curve (solid line).

Figure 6 shows the frequency shift Δf as a function of the increase of average bubble volume Δv, and its linear fit $\Delta f_{s}=2.3\times 10^{21}\Delta v+31$, where frequencies are expressed in Hz and bubble volumes in $m^{-3}$. The shift of resonance frequency, and the corresponding change of sound speed in the medium, is due to the increment of the mean bubble volume. At nonlinear regime the volume variation of the bubbles lost the symmetry around its initial volume (at rest) and underwent a displacement towards positive volume values, i.e., the bubble oscillated around a higher equilibrium volume, and the averaged volume of the bubbles rose, which induced a decrease of the sound speed.

### 3.4. Sum-Frequency Generation by Nonlinear Frequency Mixing under Nonlinear Resonance Assumption

**Figure 7:**
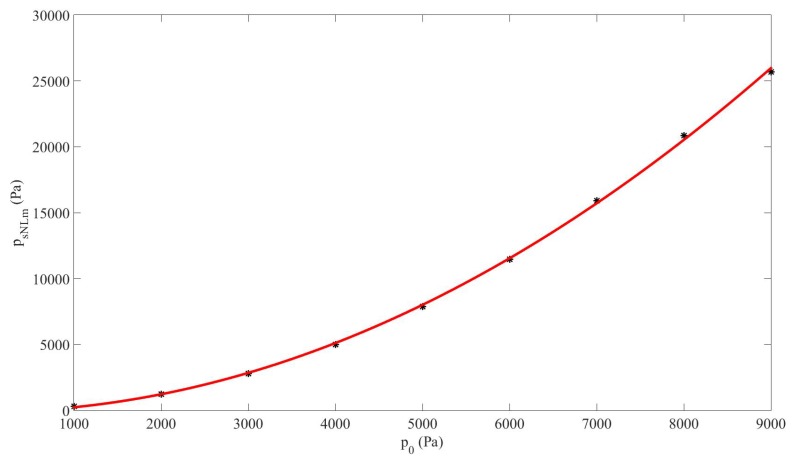
Case#2. Maximum amplitude of the sum-frequency pressure component in the cavity $p_{sNLm}$ vs. source amplitude $p_{0}$ under nonlinear resonance assumption (dots), and fitting curve (solid line).

The sum frequency obtained from Case#2, $f_{sL}=200\;\mathrm{kHz}$, by taking into account the nonlinear resonance observed in Section 3.3 was now studied. This means that we considered the “nonlinear-regime” sound speed, $c_{NL}$, at this sum frequency deduced from the value of the nonlinear resonance in the cavity of constant length found in Section 3.3: $c_{fsNL}=2Lf_{sm}=1210.2\;m/s$. We studied the generation of this sum-frequency in the cavity as a function of the source amplitude $p_{0}$. Figure 7 shows the maximum amplitude of the sum-frequency pressure component in the cavity, $p_{sNLm}$, vs. $p_{0}$ (see Figure 4). The result fits to the second-order polynomial $p_{sNLm}=3.2\times 10^{-4}p_{0}^{2}+0.03p_{0}-1.1\times 10^{-2}$, where pressures are expressed in Pa. The clear nonlinear generation of the component $f_{sNL}$ vs. $p_{0}$ indicates that its amplitude rose rapidly for all ranges of $p_{0}$, since the second-order coefficient in the polynomial was positive, $3.2\times 10^{-4}p_{0}^{2}$, which proves the efficiency of the generation for high $p_{0}$ values. The relative increases of amplitude generation of the component $f_{sL}$ in relation to $p_{0}$ were very high. For example, the increase was around 285.3% when $p_{0}=9\;\mathrm{kPa}$.

## 4. Discussion

The results obtained in Section 3 in a dispersive and nonlinear medium made of liquid and gas bubbles suggest that the generation of a higher frequency-component signal from a lower-frequency source is more efficient by nonlinear mixing of two signals from a dual-frequency source than by nonlinear cascade forming harmonics from a single-frequency source (Figure 2). It must be noted that the sum frequency from two signals with near frequency values obtained by nonlinear mixing is close to the frequency of the second harmonic formed from a single frequency. It has also been shown that the response of the sum-frequency amplitude with $p_{0}$ without taking the nonlinear resonance into account follows a cubic law for which the coefficient of the third-degree term is negative (Figure 3), whereas it follows a quadratic law with positive second-degree coefficient when the nonlinear resonance is considered (Figure 7). This characteristic allows the sum frequency to attain higher values when $p_{0}$ rises in the nonlinear resonance context. This nonlinear resonance has been characterized in Figure 4 for several $p_{0}$ and by changing the sum-frequency value to localize the frequency at which the response of the system is maximum, which defines the value of the nonlinear resonance of the cavity. The decrease of this value for higher $p_{0}$, i.e., the existence of a shift of this resonance $\Delta f_{s}$ that increases fast with $p_{0}$ following a cubic law with positive cubic coefficient (Figure 5), evidences a reduction of the sound speed, and thus, a softening of the medium, which is due to the higher mean value of bubble volume at high amplitudes, as seen by observing the proportional dependence between $\Delta f_{s}$ and the average increase of bubble volume through the linear law in Figure 6.

**Figure 8:**
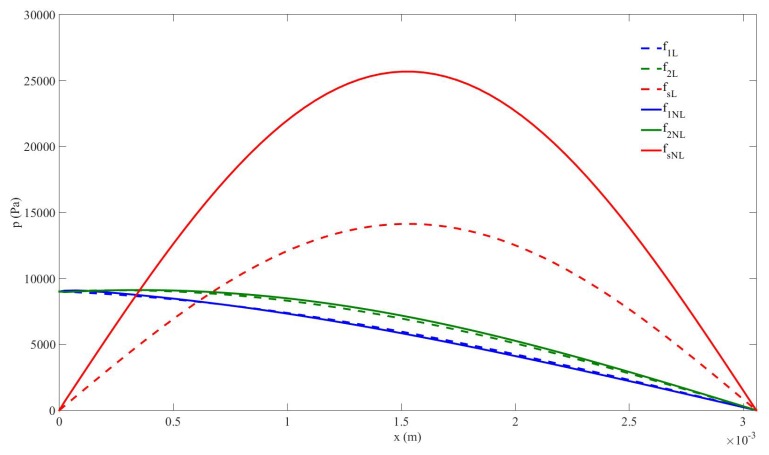
Case#2. Pressure amplitude distribution of frequency components in the cavity with $p_{0}=9\;\mathrm{kPa}$; under linear resonance assumption, $f_{1L}=90\;\mathrm{kHz}$, $f_{2L}=110\;\mathrm{kHz}$, $f_{sL}=200\;\mathrm{kHz}$ (blue, green, and red dashed lines, respectively); under nonlinear resonance assumption, $f_{1NL}=87.94\;\mathrm{kHz}$, $f_{2NL}=110\;\mathrm{kHz}$, $f_{sNL}=197.94\;\mathrm{kHz}$ (blue, green, and red solid lines, respectively).

A clear evidence of the benefits we obtain by considering the nonlinear resonance in the cavity for nonlinear mixing of two signals is through the comparison, shown in Figure 8, of the generation of the sum-frequency component obtained by accounting for the change of sound speed of the medium (solid lines, nonlinear resonance assumption) or not (dashed lines, linear resonance assumption). The same pressure-source amplitude $p_{0}=9\;\mathrm{kPa}$ is used in both configurations. When we take the nonlinear resonance effect into account the amplitude of the sum-frequency component experiments a huge increase. Its maximum value improves by 183.15%, rising from 14.016kPa (155.7% of $p_{0}$) to 25.67kPa (285.3% of $p_{0}$). The softening of the bubbly liquid when amplitudes are raised is thus an important feature of the process. It is then useful to account for this effect in applications for which the sum-frequency component needs to be enhanced.

In this work the enhancement of the generation of the sum-frequency component in bubbly liquids has been shown in a cavity by means of numerical simulations and by taking advantage of the softening of the medium at high acoustic pressure amplitudes, which has been observed through the nonlinear shift of the resonance of the cavity. In spite of the numerous restrictions used to derive the differential system solved here, inherent to every approximation model of a physical problem, the results obtained suggest that the use of a maximized signal at the sum-frequency component from mixing nonlinearly two signals of lower frequencies could be useful in several practical situations for which a higher frequency signal is required to obtain a better precision, accuracy, or resolution in frameworks like sensing, nondestructive testing, or medical imaging.

More studies should be performed in the future about the maximization of the sum-frequency signal by developing models from less restrictive differential equations, by including effects such as Bjerknes forces and others, or by setting stable experimental conditions for the propagation of ultrasound in bubbly liquids. On the other hand, the continuation of this work should also focus on the usefulness of its results to improves technical applications. Also, experimental studies should be performed to corroborate the theoretical results obtained here.
